# Research on a Simplified Model of an Aluminum Vapor Chamber in a Heat Dissipation System

**DOI:** 10.3390/e22010035

**Published:** 2019-12-25

**Authors:** Shuang Han, Lixin Yang, Zihao Tian, Xiaofei Yuan, Hongyan Lu

**Affiliations:** 1 Institute of Thermal Engineering, School of Mechanical, Electronic and Control Engineering, Beijing Jiaotong University, Beijing 100044, China; 17116386@bjtu.edu.cn (S.H.); 16116371@bjtu.edu.cn (Z.T.); 17121376@bjtu.edu.cn (X.Y.); 17121368@bjtu.edu.cn (H.L.); 2 Beijing Key Laboratory of Flow and Heat Transfer of Phase Changing in Micro and Small Scale, Beijing 100044, China

**Keywords:** aluminum vapor chamber, effective thermal conductivity, thermal resistance, simplified model

## Abstract

With the rapid increase of power densities of electronic components, the traditional heat dissipation method of air forced convection has reached a heat transfer limit. As efficient phase change heat exchangers, vapor chambers have become an important guarantee for the development of high-power electronic components. Aluminum vapor chambers have become the future development trend because they are more lightweight and less expensive. In order to study the suitable simplified model of the aluminum vapor chamber in the radiating system, the testing system is established to test the thermal characteristics of the vapor chamber. First, six simplified models of the vapor chamber are proposed. Then, the thermal characteristics of the simplified models are simulated by STAR CCM+ software. Next, the error of the thermal resistance of the simplified model and the real vapor chamber is analyzed. Finally, a most suitable simplified model is obtained in the cooling system.

## 1. Introduction

**Figure 1:**
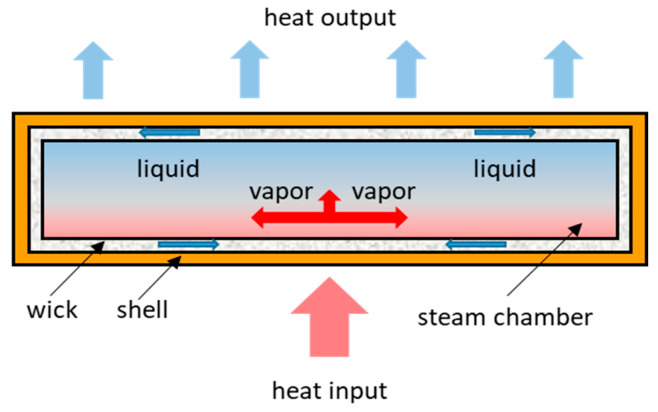
Working principle of the vapor chamber.

At present, electronic devices tend to be miniaturized and compact [1,2]. Therefore, the power density of electronic devices is increasing [3]. In order to meet the heat dissipation requirements, the cooling equipment must be efficient and flexible enough [4]. The vapor chamber is a kind of high-efficiency radiator. It is a special form of flat heat pipe [5], and is often used for thermal management of electronic devices [6]. The vapor chamber is composed of shell, wick, and steam chamber [3]. There is working fluid inside the vapor chamber. The working principle of vapor chamber is shown in Figure 1. The heat transfer process of vapor chamber is divided into four stages: (1) the working fluid absorbs heat in the evaporation surface to generate steam; (2) the steam flows to the condensation surface under the pressure difference; (3) the steam is liquefied at the condensation surface; and (4) the liquid returns to the evaporation surface under capillary force and continues to vaporize. The liquid in the wick moves continuously under the capillary pressure, which guarantees the normal operation of the vapor chamber. In addition, the temperature of the vapor chamber is more uniform. Compared to conventional heat pipes, the vapor chamber conducts heat from the heat spot in all directions without loss of efficiency [7]. Most of the vapor chambers are made of copper, which is heavy. However, the existing electronic devices are designed to be more lightweight and compact [8]. The aluminum vapor chambers, therefore, have become the future development trend due to their light weight.

In recent years, the research on vapor chambers included theoretical research [9,10,11,12], numerical simulation research [9,10,11,12], and experimental research [7,13,14,15,16,17]. The experimental research focused on the manufacture of new type vapor chambers and the performance testing of vapor chambers, which included the influence of the wick, the working fluid, the charging ratio, the heating power, and tilt angle on the thermal performance of the vapor chambers. Liu et al. [7] manufactured the vapor chamber with foamed copper as the wick. The influence of the different charging ratios, the porosity, the heat loads, and the tilt angles on the thermal performance of the vapor chambers were studied separately. Chen et al. [13] used the wick of Ω-shaped reentrant micro-channels to manufacture the vapor chamber and applied it to the thermal management of light emitting diodes (LEDs). At the same time, the thermal performance of the vapor chamber under different LED input powers, air velocity, and tilt angles was evaluated. Zeng et al. [14] studied the starting performance, thermal resistance, and temperature distribution of the aluminum vapor chamber with the micro-grooved with reentrant cavity array (MGRA) wick. The effects of heating loads, tilt angles, and cooling flow rates on the performance of the vapor chamber were analyzed. Naphon et al. [15] used the vapor chamber with refrigerant R-141b as the working fluid to cool the disk drive, and compared with the performance of the vapor chamber with water as the working fluid. It was found that the cooling performance of the vapor chamber with refrigerant R-141b as the working fluid was better. Attia et al. [16] studied the thermal performance of the vapor chamber with water and methyl alcohol as working fluids under different charging ratios. In addition, it was concluded that the performance of the vapor chamber with water as the working fluid was better than that of methyl alcohol as the working fluid. It was found that the thermal performance of the vapor chamber was the best when the charging ratio was 30%. He et al. [17] used photolithography and ion etching to manufacture the silicon vapor chamber. The complete manufacturing process including liquid filling and vacuum extraction was introduced. In addition, the performance of the designed vapor chamber was tested.

With the development of Computational Fluid Dynamics (CFD) technology, some scholars have studied the phase change heat transfer in heat pipes, and analyzed the internal temperature fields and velocity fields to provide theoretical support for the design of heat pipes. Koito et al. [9] divided the vapor chamber model into vapor region, liquid-wick region, and wall region. In addition, the temperature, velocity, and pressure field inside the vapor chamber were obtained. At the same time, the capillary pressure required for the working fluid to circulate inside the vapor chamber was obtained. Xuan et al. [18] proposed a model to simulate the dynamic behavior and steady-state performance of the flat heat pipe based on some assumptions. Ranjan et al. [19] established a transient three-dimensional numerical model of the flat heat pipe. The effect factors such as thin liquid film evaporation and meniscus curvature were taken into account. Xiao et al. [20] developed a three-dimensional model to analyze the thermohydrodynamic behavior in the flat heat pipe. In the model, the heat transfer in the wall, the coupled heat and mass transfer at the liquid–vapor interface and the fluid flow in the vapor chamber and porous wick were considered. Sobhan et al. [21] established a two-dimensional calculation model to analyze the transient operation of the flat heat pipe. The continuity equation, momentum equation, and energy equation in the wick, the transport equation of the porous wick, and the two-dimensional heat conduction equation in the wall were solved.

However, for the numerical simulation of the heat dissipation system, it is very complex to simulate the internal situation of heat pipes. Generally, the heat pipe is simplified. The heat pipe will be simplified into a solid block with the same size as the actual heat pipe and high effective thermal conductivity to reduce the calculation. However, the simplified model ignores the heat transfer process inside the heat pipe, so there is a certain error. If the temperature distribution of the simplified model differs greatly from the temperature distribution of the actual heat pipe, it may lead to a large error to the simulation of the entire system.

A simplified model has been widely used. In addition, the specific simplified models in the literature are shown in Table 1. Elnaggar et al. [22] simulated the U-shape heat pipes radiator used for computer cooling by the finite element simulation method. The effects of the air velocity, power input and heat pipe orientation on the performance of finned heat pipes were analyzed. Wang et al. [23] analyzed the effect of the length of the evaporation section, the length of the condensation section, and the heating power on working performance of the flat heat pipe. The application of a flat heat pipe in LED heat dissipation technology was numerically simulated. Zhang et al. [24] studied the thermal performance of three-dimensional heat pipe flat solar collectors based on the finite volume method, which helped to design and optimize heat pipe flat solar collectors. Chen et al. [10] used isotropic and anisotropic methods to calculate the effective thermal conductivity of the vapor chamber. The results showed that the anisotropic method can better represent the heat transfer characteristics of the vapor chamber. Velardo et al. [11] established a two-dimensional axisymmetric model of the vapor chamber. In addition, the concept of effective thermal conductivity was introduced to calculate the thermal performance of the vapor chamber. Velardo et al. [12] improved the simplified model of the vapor chamber based on the original research. The simplified model included the wall region, vapor region, and wick region. The author mainly studied the effective thermal conductivity of the vapor region. Li et al. [25] performed the numerical simulation on the high-power LED multi-chip package module. In addition, the heat pipe was simplified into a solid block with the inherent effective thermal conductivity in the axial direction and the radial direction, respectively. It was found that the heat pipe can effectively reduce the temperature of the LED heat source. Zhang et al. [26] experimentally and numerically studied the thermal management system of the lithium battery based on the flat heat pipe. In the numerical simulation research, the flat heat pipe was simplified into a solid block with inherent effective thermal conductivity. 

Due to the small internal space, the phase change simulation of thin aluminum vapor chambers is more difficult. In the actual design of the vapor chambers’ heat dissipation system, the thin aluminum vapor chambers are also simplified as the heat pipe. However, there is little research on a suitable simplified model of the vapor chamber. In addition, the relationship between the setting of effective thermal conductivity in the simplified model and the simulation accuracy has not been explained. Therefore, this paper establishes the thermal performance testing system of the vapor chamber, and analyzes the thermal characteristics of the vapor chamber. Six simplified models of the vapor chamber are established, which are numerically simulated to obtain their thermal performance. Furthermore, the error between the simplified model and the real vapor chamber is analyzed to get the suitable simplified model, which provides significant guidance for the numerical simulation of the heat dissipation system of the vapor chamber. 

## 2. Experimental Facility 

### 2.1. Test Section 

**Figure 2:**
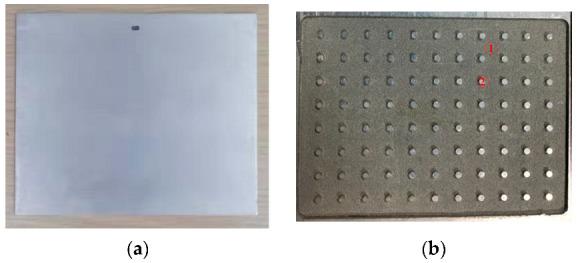
Vapor chamber. (**a**) appearance of vapor chamber; (**b**) internal structure of vapor chamber (1—Wick; 2—Supporting column).

**Figure 3:**
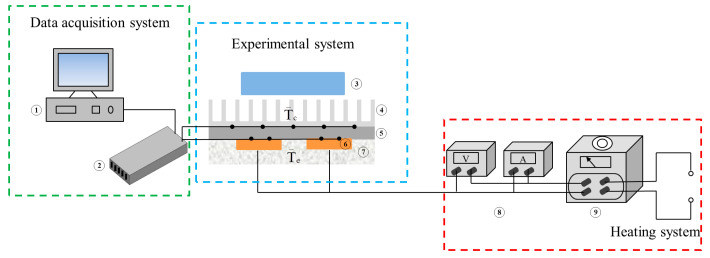
Schematic testing system. 1—Computer; 2—Data acquisition instrument; 3—Fan; 4—Fin; 5—Vapor chamber; 6—Heat block; 7—Insulation block; 8—Display; 9—Voltage regulator.

**Figure 4:**
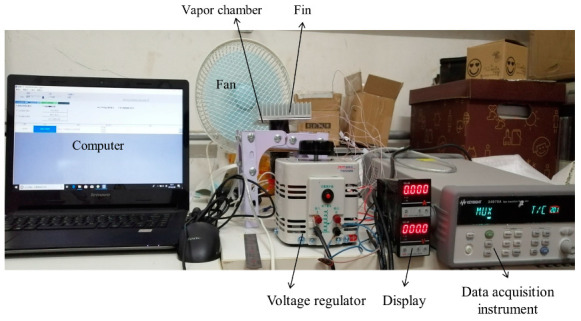
Testing system.

The size of thin aluminum vapor chambers is 120 × 100 × 2 mm, as shown in Figure 2a. The wick is aluminum powder sintered and the thickness of the wick is 0.3 mm, as shown in Figure 2b. In order to enhance the strength of the vapor chamber, a plurality of supporting columns is arranged inside the vapor chamber. The working fluid is acetone. The charging ratio is 40%. The test section consists of a data acquisition system, experimental system, and heating system. Two heating blocks are evenly located on the lower surface of the vapor chamber. The schematic diagram of the testing system is shown in Figure 3, for which the actual testing system is shown in Figure 4. The size of heat block is 30 × 25 × 1.5 mm.

### 2.2. Data Analysis

The calculation formula of the thermal resistance of vapor chambers is:

(1) $$R=\frac{\bar{T_{e}}-\bar{T_{c}}}{Q}=\frac{\bar{T_{e}}-\bar{T_{c}}}{UI}$$

R is the thermal resistance of vapor chamber, Q is the heat power, U is the input voltage, I is the input current, $\bar{T_{e}}$ is the average temperature of the evaporation surface, and $\bar{T_{c}}$ is the average temperature of the condensation surface.

**Figure 5:**
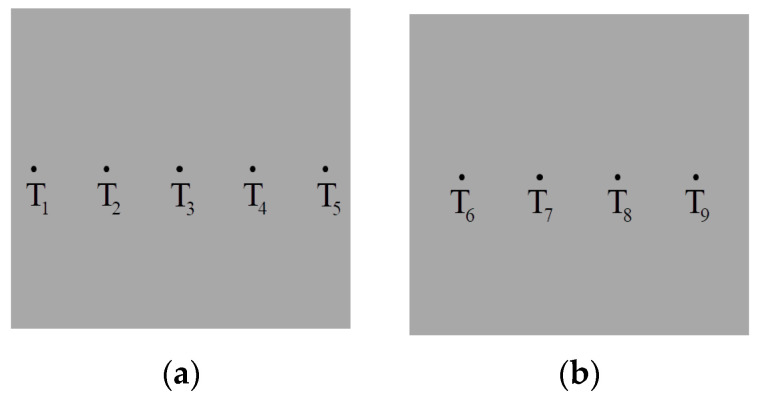
Thermocouple location distribution. (**a**) condensation surface; (**b**) evaporation surface.

The distribution of thermocouples of evaporation and condensation surface is shown in Figure 5. Five thermocouples are evenly placed on the center line of the interface between the condensation surface and the fin. Similarly, two thermocouples are evenly arranged on the center line of the interface between the evaporation surface and each heating block. 

The average temperature of the evaporation surface is the average of the four thermocouples at the interface between the evaporation surface and the heating blocks. The average temperature of the condensation surface is the average of the five thermocouples at the interface between the condensation surface and the fins.

The calculation formulas of the average temperature are:

(2) $${\bar{T}}_{e}=\left(T_{6}+T_{7}+T_{8}+T_{9}\right)/4,$$

(3) $${\bar{T}}_{c}=\left(T_{1}+T_{2}+T_{3}+T_{4}+T_{5}\right)/5.$$

The calculation formula of the effective thermal conductivity of vapor chambers is:

(4) $$k_{e}=\frac{\Delta h}{AR}.$$

$k_{e}$ is the effective thermal conductivity, Δh is the thickness of vapor chambers, and A is the cross-sectional area of vapor chambers.

### 2.3. Uncertainty Analysis

The uncertainty of experimental data is calculated by error transfer theory [27]. If *F* is a function of the independent parameter *X*, *Y*, *Z*, and Δ*X*, Δ*Y*, Δ*Z* is the uncertainty of each independent parameter, then the uncertainty of F is:

(5) $$\Delta F=\sqrt{{\left[\left(\partial F/\partial X\right)\Delta X\right]}^{2}+{\left[\left(\partial F/\partial Y\right)\Delta Y\right]}^{2}+{\left[\left(\partial F/\partial Z\right)\Delta Z\right]}^{2}+\cdots }.$$

The uncertainty of the thermal resistance of vapor chambers is:

(6) $$\Delta R=\pm \sqrt{{\left(\Delta T/T\right)}^{2}+{\left(\Delta U/U\right)}^{2}+{\left(\Delta I/I\right)}^{2}}.$$

The uncertainty of the main parameters in the experiment is shown in Table 2.

### 2.4. Experimental Result

**Figure 6:**
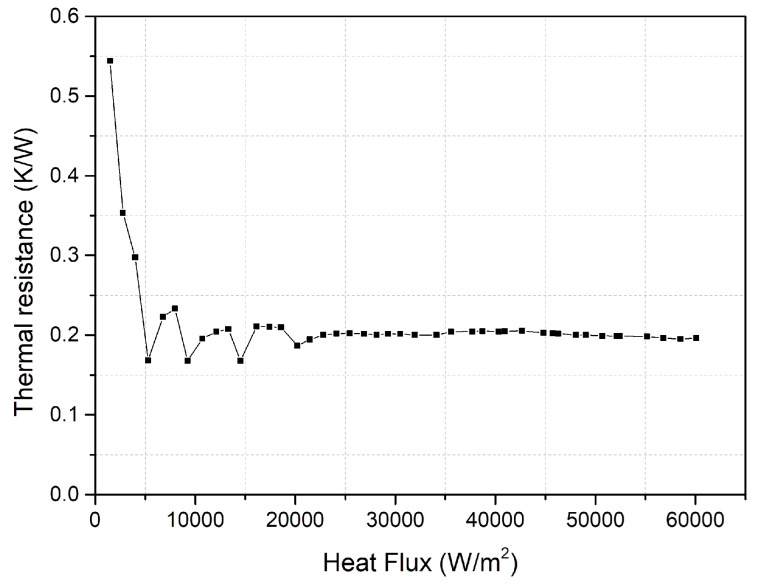
Thermal resistance of the vapor chamber.

Figure 6 shows the thermal resistance of vapor chamber at different heat flux. When the heat flux is relatively low, the vapor chamber has not fully started to work because the temperature has not reached the boiling point of the working fluid. Thus, the thermal resistance of the vapor chamber at this time is relatively high. As the heat flux increases, the working fluid in the vapor chamber undergoes phase change because the boiling point of the working fluid is reached. Therefore, the thermal resistance of the vapor chamber decreases. Since the maximum operating temperature of the electronic components is 80 °C, the experiment stops when the temperature of evaporation surface reaches 80 °C. It can be seen from Figure 6 that, when the vapor chamber works normally and the temperature of evaporation surface is less than 80 °C, the average thermal resistance of the vapor chamber is a constant value of 0.2 K/W, and there is no dry-out phenomenon. Therefore, using vapor chamber heat dissipation for electronic components is a good thermal management method.

## 3. Analysis and Modeling

**Figure 7:**
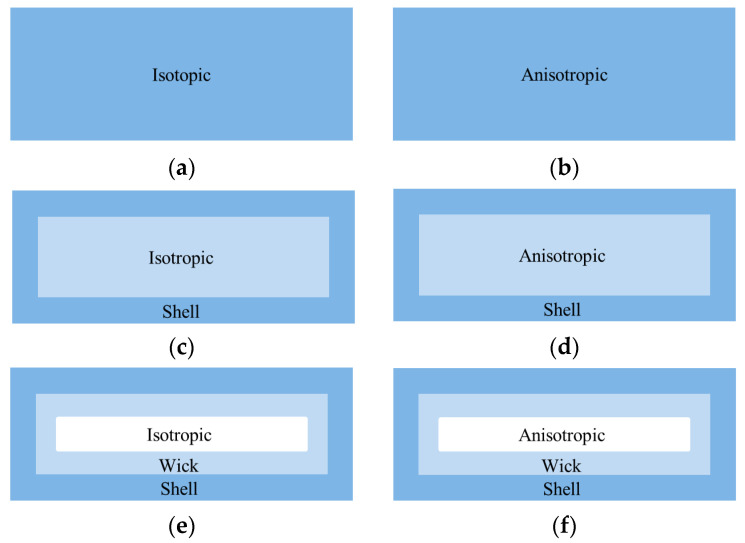
Simplified models of vapor chamber. (**a**) Model 1; (**b**) Model 2; (**c**) Model 3; (**d**) Model 4; (**e**) Model 5; (**f**) Model 6.

According to the existing literature and actual research, the simplified models should be the same size as the actual vapor chamber, and the simplified models are all solid blocks with fixed thermal conductivity. There are six simplified models for the vapor chamber: (1) isotropic solid block; (2) anisotropic solid block; (3) splitting the vapor chamber into the shell and the other part. The shell is aluminum alloy, and the other part is the isotropic solid block; (4) splitting the vapor chamber into the shell and the other part. The shell is aluminum alloy, and the other part is anisotropic solid block; (5) splitting the vapor chamber into the shell, wick, and steam chamber. The shell is the aluminum alloy. The wick is a solid block, and the steam chamber is an isotropic solid block; and (6) splitting the vapor chamber into the shell, wick, and steam chamber. The shell is the aluminum alloy. The wick is a solid block, and the steam chamber is an anisotropic solid block. The simplified models are shown in Figure 7.

### 3.1. Geometric Model

**Figure 8:**
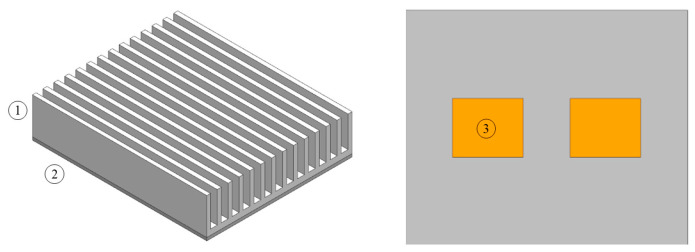
Geometric model. 1-Fin; 2-Vapor chamber; 3-Heat block.

The geometric model is shown in Figure 8. The geometric model includes fin, vapor chamber, and heat blocks.

### 3.2. Mesh Model

**Figure 9:**
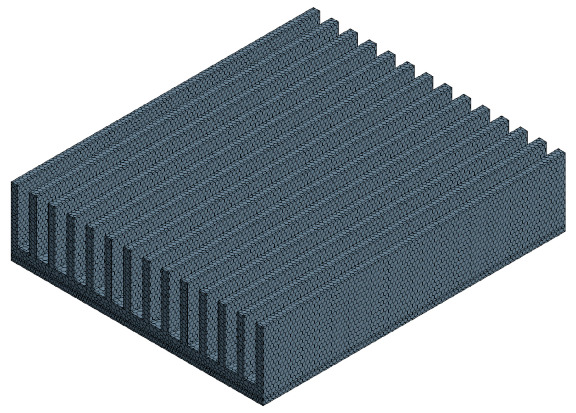
Mesh model.

The mesh model is surface reconstruction and polyhedral mesh. Taking Model 2 and heat flux density as 1476.8 W/m^2^ as an example, the grid sensitivity analysis is shown in Table 3. Considering the number of grids and the stability of the calculation results, the grid base size of 2 mm is finally selected. The mesh is shown in Figure 9.

### 3.3. Physical Model

The energy equation is:

(7) $$k(\frac{\partial^{2}T}{\partial x^{2}}+\frac{\partial^{2}T}{\partial y^{2}}+\frac{\partial^{2}T}{\partial z^{2}})=0.$$

Boundary conditions include heating surfaces, fin surface, and other surfaces. 

Heating surfaces are:

(8) $$-k\frac{\partial T}{\vec{n}}=q.$$

Fin surface is:

(9) $$-k\frac{\partial T}{\vec{n}}=h({\bar{T}}_{c}-T_{f}).$$

The other surfaces are:

(10) $$-k\frac{\partial T}{\vec{n}}=0.$$

k is the thermal conductivity, T is the temperature, q is the heat flux, h is the convective heat transfer coefficient, and $T_{f}$ is the ambient temperature.

**Figure 10:**
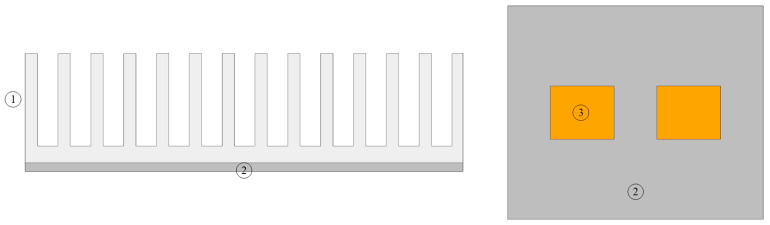
The boundary conditions of the model. 1—Fin surface; 2—Other surfaces; 3—Heating surfaces.

The boundary conditions are shown in Figure 10.

When the flow is laminar, the convective heat transfer coefficient of fin is:

(11) $$Nu=\frac{hL_{f}}{k_{a}}=0.664Re^{0.5}Pr^{1/3}.$$

When the flow is turbulent, the convective heat transfer coefficient of fin is:

(12) $$Nu=\frac{hL_{f}}{k_{a}}=0.037Re^{0.8}Pr^{1/3},$$

(13) $$Re=\frac{VL_{f}}{\nu }.$$

Nu is Nusselt number, h is the convective heat transfer coefficient, $L_{f}$ is the length of fin, $k_{a}$ is the thermal conductivity of air, Re is Reynolds number of air, Pr is Prandtl number of air, V is the velocity of air, and ν is Kinematic viscosity coefficient of air.

The effective thermal conductivity of the vapor chamber contains the effective thermal conductivity in the X, Y, and Z directions and the effective thermal conductivity of the wick:

(14) $$k_{w}=k_{s}\left[\frac{2+k_{l}/k_{s}-2\cdot \varepsilon \cdot (1-k_{l}/k_{s})}{2+k_{l}/k_{s}+\varepsilon \cdot (1-k_{l}/k_{s})}\right],$$

(15) $$k_{X}=\frac{L_{X}}{A_{X}R},$$

(16) $$k_{Y}=\frac{L_{Y}}{A_{Y}R},$$

(17) $$k_{Z}=\frac{L_{Z}}{A_{Z}R}.$$

$k_{w}$ is the effective thermal conductivity of the wick, $k_{l}$ is the thermal conductivity of the liquid in the wick, $k_{s}$ is the thermal conductivity of the solid material of the wick, and ε is the porosity of the wick. $k_{X}$, $k_{Y}$, and $k_{Z}$ are respectively the effective thermal conductivity of the vapor chamber in X, Y, and Z directions, $L_{X}$, $L_{Y}$, and $L_{Z}$ are respectively the length of the vapor chamber in X, Y, and Z directions, $A_{X}$, $A_{Y}$, and $A_{Z}$ are respectively the cross-sectional area of the vapor chamber in X, Y, and Z directions, and R is the average thermal resistance of vapor chamber when the vapor chamber works normally. In the numerical simulation calculation, it is assumed that the vapor chamber is working normally.

**Figure 11:**
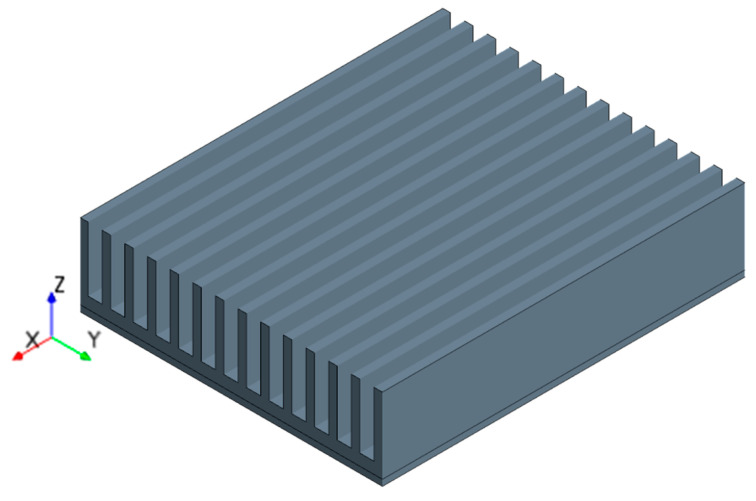
X, Y, and Z directions of simplified models.

In order to estimate the effective thermal conductivity for wick, Maxwell has presented Equation (14) that offers the thermal conductivity of such a heterogeneous material [28]. The effective thermal conductivity of the vapor chamber in the X, Y, and Z directions is calculated as Equations (15)–(17). X, Y, and Z directions of simplified models is shown in Figure 11.

The effective thermal conductivity of different models is set as shown in Table 4.

## 4. Results and Discussion

### 4.1. Results Analysis

The temperature distribution of the simplified models under different heat flux densities is obtained. The temperature of simplified model corresponding to the position of thermocouples in the experiment is taken out to obtain the average temperature of the evaporation surface and the condensation surface in the simulation. Thus, the thermal resistance of the simplified models is obtained according to Equation (1).

**Figure 12:**
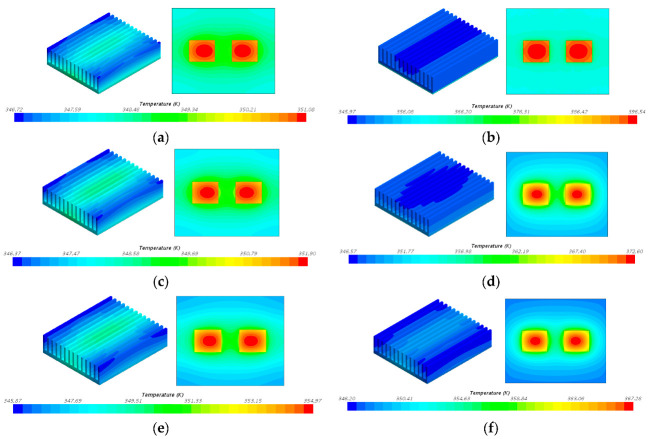
Temperature field cloud pictures of six models when the heat flux is 60,000 W/m^2^. (**a**) Model 1; (**b**) Model 2; (**c**) Model 3; (**d**) Model 4; (**e**) Model 5; (**f**) Model 6.

The temperature field cloud pictures of six models are shown in Figure 12 when the heat flux is 60,000 W/m^2^. Table 5 is thermal resistance of different simplified models in numerical simulation. When the model is simplified as an isotropic solid block, the thermal resistance values are all low. Therefore, it is not appropriate to simplify the vapor chamber in the cooling system to a model with high thermal conductivity in all directions. 

The accuracy of the simplified model is judged based on the relative error of the experimental and simulated thermal resistance:

$$\mathrm{Relative}\;\mathrm{error}=\frac{\left|\mathrm{Simulated}\;\mathrm{value}-\mathrm{Experimental}\;\mathrm{value}\right|}{\mathrm{Experimental}\;\mathrm{value}}\times 100\%.$$

**Figure 13:**
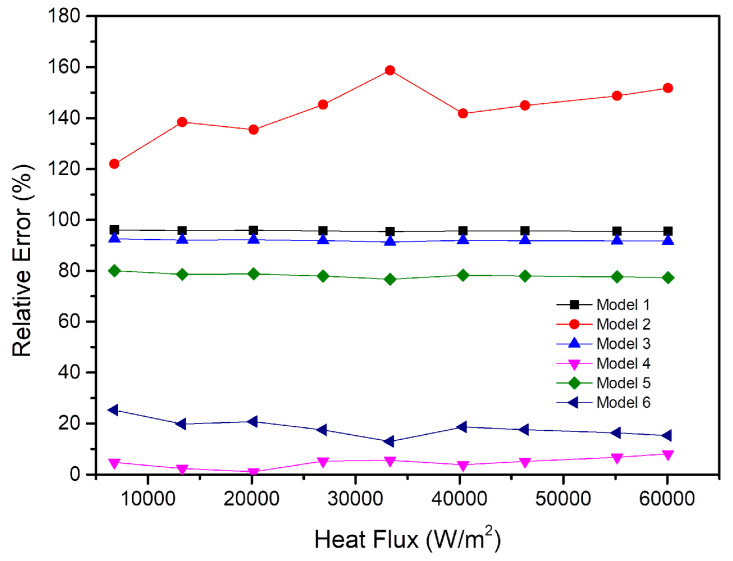
Relative errors of the six models.

The relative errors of the six simplified methods under different heat flux are shown in Figure 13.

As shown in Figure 13, the results of simplifying the vapor chamber into the shell and the other part are better than the results of other simplified models. Model 1, Model 3, and Model 5 simplify the vapor chamber to containing an isotropic solid block with a high thermal conductivity, which results in a low thermal resistance for the three models. However, in fact, the thermal conductivity in the X direction, Y direction, and Z direction of the vapor chamber is not the same, and the effective thermal conductivity in the Z direction is much smaller than that value in the X direction and the Y direction. Though the relative errors of Model 4 and Model 6 are both lower than 30%, the relative error of Model 6 is higher than that of Model 4. Meanwhile, the thermal resistance of Model 6 is lower than the experimental results, which is unfavorable for the design of the heat dissipation system. Therefore, the simplified Model 4 is more suitable than the other simplified models. 

### 4.2. Results Verification

**Figure 14:**
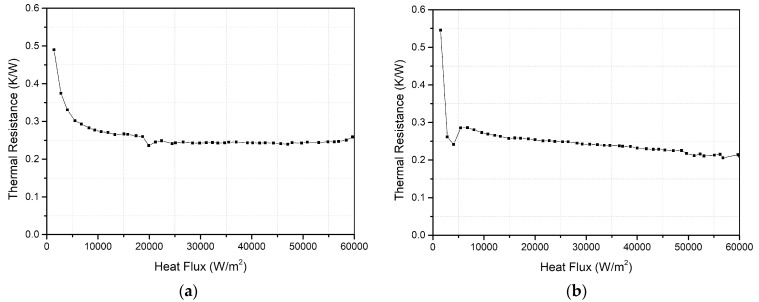
Thermal resistance of the vapor chamber. (**a**) the charging ratio is 50% and the number of heating blocks is two; (**b**) the charging ratio is 50% and the number of heating blocks is three.

**Figure 15:**
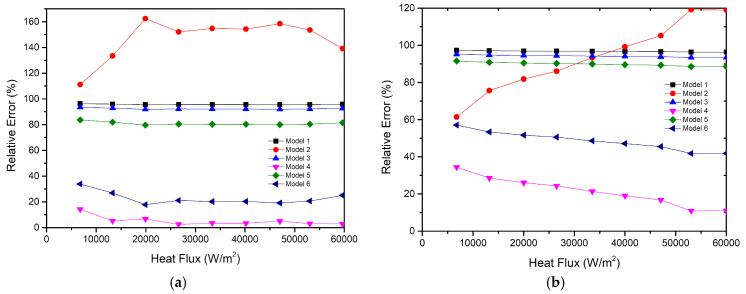
Relative errors of the six models. (**a**) the charging ratio is 50% and the number of heating blocks is two; (**b**) the charging ratio is 50% and the number of heating blocks is three.

Since the above results are based on charging ratio of 40% and two heating blocks, it can’t represent a general conclusion. Therefore, two other test conditions are selected: (1) the charging ratio is 50%, two heating blocks; (2) the charging ratio is 50%, three heating blocks. The experimental results are shown in Figure 14. In addition, the relative errors of the different models are shown in Figure 15.

When the charging ratio is 50% and the number of heating blocks is two, the average thermal heat resistance of the vapor chamber is 0.25 K/W, and the simplified Model 4 is the most suitable. When the charging ratio is 50% and the number of heating blocks is three, the average thermal heat resistance of the vapor chamber is 0.24 K/W. Although the relative errors under this condition are larger than the relative errors under other conditions, the average relative errors of Model 4 are lower than 30%. Therefore, it can still be found that simplified Model 4 is the most appropriate. 

## 5. Conclusions

In this paper, a testing system for the thermal performance of the vapor chamber is established. First, the thermal resistance and effective thermal conductivity of the vapor chamber is obtained. Then, the simplified model of the vapor chamber applied to the heat dissipation system is proposed. Next, the simplified model of the vapor chamber is numerically simulated to obtain a suitable simplified model. Finally, the following conclusions can be drawn:

(1) As the heat flux density increases, the thermal resistance of the vapor chamber is smaller and the thermal performance is better. Within the working temperature range of electronic components, the thermal resistance of the vapor chamber is a constant value of 0.2 K/W, and there is no dry-out phenomenon.
(2) By numerically simulating the six simplified models, the thermal resistance of the six models is analyzed. Then, the conclusion that simplifying the vapor chamber into the shell and the other part is suitable compared to other models is finally obtained.
(3) Although the simplified Model 4 is the most suitable model of the vapor chamber under different test conditions, the results show that the accuracy of the model decreases with more heat sources.
(4) By analyzing simplified models of the vapor chamber under different testing conditions, the most suitable and general simplified Model 4 in the heat dissipation system is obtained, which is very significant for industrial thermal design.

## Figures and Tables

**Table 1:** Simplified models in the literature.

Simplified Models	Reference
Anisotropic entire region	[10,24,26]
Isotropic entire region	[11,23,25,27]
The entire region divided into the wall region, vapor region, and wick region	[12]

**Table 2:** Uncertainty of main parameters.

Measured Value		Calculated Value	
Parameter	Uncertainty	Parameter	Uncertainty
T	±0.25 K	R	±1.47%
U	±1%		
I	±1%		

**Table 3:** The grid sensitivity analysis.

Base Size of the Grid	Number of the Grid	Temperature Difference between Evaporation and Condensation Surface	Thermal Resistance
1 mm	21,6870	1.104300	0.498510293
2 mm	130,371	1.095600	0.494582882
4 mm	123,065	1.141900	0.515483929
8 mm	37,517	1.572700	0.709958469

**Table 4:** The effective thermal conductivity of different models.

Model	Shell	Wick	Steam Chamber
Model 1	$k_{X}$ in all directions		
Model 2	$k_{X}$ in X directions, $k_{Y}$ in Y directions, $k_{Y}$ in Y directions		
Model 3	Aluminum alloy	$k_{X}$ in all directions	
Model 4	Aluminum alloy	$k_{X}$ in X directions, $k_{Y}$ in Y directions, $k_{Y}$ in Y directions	
Model 5	Aluminum alloy	$k_{w}$	$k_{X}$ in all directions
Model 6	Aluminum alloy	$k_{w}$	$k_{X}$ in X directions, $k_{Y}$ in Y directions, $k_{Y}$ in Y directions

**Table 5:** Thermal resistance of different models in numerical simulation.

Model	Thermal Resistance
model 1	0.0088
model 2	0.4946
model 3	0.01651
model 4	0.2123
model 5	0.04451
model 6	0.16644
